# Impacts of night shift on medical professionals: a pilot study of brain connectivity and gut microbiota

**DOI:** 10.3389/fnins.2025.1503176

**Published:** 2025-02-17

**Authors:** Tengmao Yao, Yi-Ping Chao, Chih-Mao Huang, Hsin-Chien Lee, Chi-Yun Liu, Kuan-Wei Li, Ai-Ling Hsu, Yu-Tang Tung, Changwei W. Wu

**Affiliations:** ^1^Graduate Institute of Mind, Brain and Consciousness, Taipei Medical University, Taipei, Taiwan; ^2^Department of Computer Science and Information Engineering, Chang Gung University, Taoyuan, Taiwan; ^3^Department of Otolaryngology-Head and Neck Surgery, Chang Gung Memorial Hospital at Linkou, Taoyuan, Taiwan; ^4^Department of Biological Science and Technology, National Yang Ming Chiao Tung University, Hsinchu, Taiwan; ^5^Center for Intelligent Drug Systems and Smart Bio-devices (IDS2B), National Yang Ming Chiao Tung University, Hsinchu, Taiwan; ^6^Graduate Institute of Humanities in Medicine, College of Humanities and Social Sciences, Taipei Medical University, Taipei, Taiwan; ^7^Research Center of Sleep Medicine, Taipei Medical University Hospital, Taipei, Taiwan; ^8^School of Nutrition and Health Sciences, Taipei Medical University, Taipei, Taiwan; ^9^National Center for Geriatrics and Welfare Research, National Health Research Institutes, Yunlin, Taiwan; ^10^Department of Artificial Intelligence, Chang Gung University, Taoyuan, Taiwan; ^11^Department of Psychiatry, Chang Gung Memorial Hospital at Linkou, Taoyuan, Taiwan; ^12^Graduate Institute of Biotechnology, National Chung Hsing University, Taichung, Taiwan; ^13^Cell Physiology and Molecular Image Research Center, Wan Fang Hospital, Taipei Medical University, Taipei, Taiwan

**Keywords:** shift work, attention, circadian rhythm, functional MRI, brain connectivity, gut microbiota, gut-brain axis

## Abstract

Night shift is a prevalent workstyle in medical hospitals, demanding continuous health monitoring and rapid decision making of medical professionals. Night shifts may cause serious health problems to medical staff, including cognitive impairments, poor sleep, and inflammatory responses, leading to the altered gut-brain axis. However, how night shifts impact gut-brain axis and how long the impact lasts remain to be studied. Hence, we investigated the dynamic changes of brain-microbiota relations following night shifts and subsequent recovery days among medical shift workers. Young medical staffs were recruited for the 3-session assessments over the scheduled night shifts (pre-shift, post-shift, and recovery) by measuring (a) sleep metrics, (b) brain functions, (c) gut bacteriome compositions, and (d) cognitive assessments. Participants experienced partial sleep deprivation only during the 5-day night shifts but rapidly returned to baseline after the 4-day recovery, so as the elevated brain fluctuations in the superior frontal gyrus after night shifts. Meanwhile, the night shifts caused elongated connectivity changes of default-mode and dorsal attention networks without recovery. Nevertheless, we did not find prevailing night-shift effects on cognition and gut bacteriome compositions, except the *Gemellaceae* concentration and the multi-task performance. Collectively, night shifts may induce prolonged alterations on brain connectivity without impacts on gut bacteriome, suggesting the vulnerable brain functions and the resilient gut bacteriome to the short-term night shifts among medical shift workers.

## Introduction

Shift work, the irregularly arranged work schedule, is prevailing among the specific job types requiring consecutive surveillance and swift decision making, such as the road constructor, medical caregiver, and aircraft traffic controller (Machi et al., 2012; Mélan and Cascino, 2022). It was reported that 15 million American and 1.5 million Australian are employed in a working style of night shifts or rotating shifts (Rajaratnam et al., 2013; Rogers et al., 2021), and the shift workers are generally suffered from the circadian misalignment, poor sleep quality, chronic fatigue and higher risk of dementia (Bokenberger et al., 2018; Gilavand, 2022). In Taiwan, the Taiwan Society of Sleep Medicine (TSSM) reported the prevalence of chronic insomnia among the Taiwanese shift workers was about 23.3%, about 2.18 times higher than the daytime workers (TSSM, 2019). In the Taiwanese medical field, the consecutive rotation of 3–5-night shifts in a row are a common schedule across medical specialties (Chang et al., 2017); however, the prolonged wakefulness and diminished vigilance during the circadian trough easily led the medical staffs to poor performances or even medical errors (Kalmbach et al., 2017; Kang et al., 2020; Qiu et al., 2020). The most prominent and subject drawback of the night shift is the attention impairment, and other domains of cognitive functions are also affected, such as the decision making, emotion and memory (Kecklund and Axelsson, 2016). During the COVID-19 outbreak, the bursts of workloads further elevated the shift-work burdens to the clinical staffs, causing inevitable burnout and fatigue as the results (Xia et al., 2021; Gilavand et al., 2023). These aversive effects of medical shift work not only impact the quality of patient healthcare, but also a risk factor of traffic accident and work-life balance for the medical staffs.

Underlying the cognitive impairments and behavioral risks, previous literature has addressed that the night shift causes various detrimental effects on biology, such as the melatonin depletion, impaired glucose homeostasis, increased pro-inflammatory cytokines and oxidative stress (Kecklund and Axelsson, 2016; Wu et al., 2021). However, such biomarkers could cause the aversive effect on physical health, but indirect to the cognitive impairments following night shifts. The direct cause of the cognitive decline after night shifts is speculated as the neurophysiological factors, such as the brain functions and gut microbial functions. From the angle of brain functions, the shift work may induce abnormal brain functionality (Wu et al., 2021; Tian et al., 2022), regional cerebral blood flow (Park et al., 2019), and decreased cerebrospinal fluid (CSF) volume, leading to the alterations of plasma *β*-amyloid and tau protein (Ye et al., 2023). For medical staffs, shift works associated with the reduction of long-range functional connectivity in the medial frontal gyrus (part of default-mode network, DMN) (Ye et al., 2022), and lower brain activity/connectivity in the right dorsal attention network (DAN) (Dong et al., 2024). Meanwhile, the gut microbiota and their corresponding metabolites are regarded as the second central nervous system involved in the cognitive performances as well (Mashaqi and Gozal, 2020). In the animal model, abundant evidence disclosed that the circadian-rhythm misalignment could lead to the microbial malfunctions, such as the decreased proportion of *Firmicutes* and the elevated abundance of *Proteobacteria* at the phylum level (Hu et al., 2022), involved in dysfunctional glucose homeostasis (Altaha et al., 2022). However, even though literature discloses the key role of the gut-brain axis in cognition and circadian rhythms (Cryan and Dinan, 2012; Teichman et al., 2020; Cai et al., 2021), the quantifiable relationship between brain functions and gut microbial compositions remains to be investigated for shift workers.

Rather than taking place as a switch, the medical staffs usually undergo night shifts in a long-term rotation, which lead to worse cognitive performances (Machi et al., 2012; Sun et al., 2021), and the cognitive decline might not return to the baseline after years (Marquié et al., 2015). Such chronic effect of shift work induces additional question: How many resting days after the night shifts would be sufficient to alleviate the aversive effects of cognitive and neurophysiological impairments? Previously, Belenky et al. (2003) exhibited that the 3 days of normal sleep following a 7-day sleep restriction were insufficient to restore the cognitive impairments, and Brown et al. (2020) also showed that the reduced sleep efficiency during the night shifts might return to normal after 5 days of recovery. From such evidence, it is reasonable to speculate the aversive effects of night shift could prolong as an inertia effect for the consecutive few days with normal circadian rhythms, which awaits to be investigated as well.

Altogether, the research questions intrigued the following two hypotheses: (1) night shifts in medical professionals can lead to alterations of brain functions, gut microbial compositions, and their mutual relations; (2) night-shift-induced changes of brain function and gut microbiota may not quickly recover after few days of normal circadian rhythms. To probe the gut-brain relationship, we conducted a functional magnetic resonance imaging (fMRI) protocol to assess the brain functionality, collected the fecal sample for assessing the gut bacteriome composition and their metabolites, short-chain fatty acids (SCFA), and probed the cognitive performances and sleep metrics among Taiwanese medical staffs in a repeated-measure design (3 time points: pre-shift, post-shift, recovery).

## Materials and methods

### Participants

We recruited 15 medical personnel from hospitals in the Taipei metropolitan area, including registered nurses, radiologists, and pharmacists. Participants eligible for the study should meet the following inclusion criteria: (1) age between 20 and 65 years; (2) without any neurological or psychiatric disorders; (3) no history of addictive drug use or habitual alcohol consumption; (4) no antibiotics, probiotics, prebiotics, or antifungal medications for 3 months prior to experiment; (5) not taking any sleeping aids in the past 2 weeks; (6) without pregnancy; (7) without any metal implants incompatible with Magnetic Resonance Imaging (MRI), such as pacemakers, metal pins, or cardiac stents; (8) without claustrophobia; and (9) without any type of intestinal pathogens. The participants were recruited for having a normal schedule of consecutive shift work for at least 4 days with a subsequent three-day resting period after shifts, and all participants provided written informed consents. Procedures in this experiment were approved by the Taipei Medical University - Joint Institutional Review Board (TMU-JIRB N202105081), and we confirmed that all methods in this study were performed in accordance with the relevant guidelines and regulations.

### Experimental procedure

All participants were instructed to visit the MRI center for three times to assess the effects of shift work in a repeated-measure design. The three time points contained (a) before starting their shift work (*pre-shift*), (b) after working in night shift for at least 4 days (*post-shift*), and (c) at least 3 days of back-to-normal-sleep after shift days (*recovery*). We held an experimental briefing 7 days before each participant’s first fMRI experiment. All participants were required to wear actigraphy devices (Philips Respironics Inc., Pittsburgh, Pennsylvania) to measure their sleep metrics, including total sleep time (TST), sleep efficiency (SE), and wake after sleep onset (WASO).

### Data collection: fecal sample, fMRI protocol and cognitive tasks

The MRI experiments were scheduled before 2 pm to fulfill the participants’ off-work times and to avoid circadian trough. On the scanning date, participants were requested to avoid consuming any beverages containing caffeine or alcohol 24 h prior to the experiment. In the morning before the scheduled MRI scanning, participants were instructed to collect fecal samples (1 g) properly using the DNA/RNA ShieldTM Fecal Collection Tube (PANGEA laboratory, USA). Fecal samples were stored in tubes containing DNA stabilization buffer to prevent DNA degradation at room temperature. We conducted the MRI experiments at National Taiwan University with a 3T PRISMA scanner (Siemens, Erlangen, Germany) using a 20-channel birdcage head coil. The scanning protocol included one 3D-MPRAGE T_1_-weighted anatomical image and a resting-state fMRI scan. Parameters for the anatomical 3D-MPRAGE sequence: image dimensions = 256 × 256 × 192; voxel size = 1 × 1 × 1 mm^3^; repetition time (TR) = 2 s, echo time (TE) = 2.3 msec; flip angle (FA) = 8°; total scan time = 6 min 24 s. The fMRI scans were using single-shot gradient-echo-based echo-planar imaging (GE-EPI) sequence with the following imaging parameters: image dimensions = 64 × 64 × 33; voxel size = 3.44 × 3.44 × 3.4 mm^3^; TR = 2 s, TE = 32 msec, FA = 77°. The total scan time for resting-state fMRI was 7 min.

After the fMRI experiment, we administered the Cambridge Neuropsychological Test Automated Battery (CANTAB) on an iPad (Apple Inc., California, US) to investigate the overall cognitive performances. We examined four cognitive domains of the CANTAB tasks, including (A) *Memory function*, including 3 tasks: delayed matching sample (DMS), paired associates learning (PAL), and spatial span (SSP); (B) *Psychomotor ability*, including reaction time (RTI) task; (C) *Emotion and social recognition function*, including emotion recognition task (ERT); and (D) *Executive function*, including multitasking test (MTT), spatial working memory (SWM), and stop signal task (SST). The time to complete the entire CANTAB examinations was approximately 1 h.

### fMRI preprocessing and analyses

SPM 12 and CONN toolbox were used for image preprocessing, seed-based connectivity analysis, and region of interest (ROI) analysis. Functional and anatomical data were preprocessed using a flexible preprocessing pipeline including smoothing. Anatomical data were normalized into standard MNI space, segmented into grey matter, white matter, and CSF tissue classes, and resampled to 1-mm isotropic voxels using SPM unified segmentation and normalization algorithm with the default IXI-549 tissue probability template. Functional data were smoothed using spatial convolution with a Gaussian kernel of 6 mm full-width half maximum (FWHM). In addition, functional data were denoised using a standard denoising pipeline including the regression of potential confounding effects characterized by white matter, CSF, motion parameters and their first order derivatives, outlier scans, and linear trends (2 factors) within each functional run, followed by bandpass frequency filtering of the BOLD time series between 0.008 Hz and 0.09 Hz. From the number of noise terms included in this denoising strategy, the average degrees of freedom of the BOLD signal after denoising were estimated to be 482.3 (range from 182.4 to 549.7) across all subjects.

Two indices of brain functionality were chosen to estimate the shift-work effects: amplitude of low-frequency fluctuation (ALFF) and seed-based connectivity (SBC) analysis to estimate the patterns of spontaneity fluctuations and functional connectivity (FC), respectively, within the 164 ROIs of HPC-ICA and Harvard-Oxford templates (Whitfield-Gabrieli and Nieto-Castanon, 2012). FC strength was represented by Fisher-transformed bivariate correlation coefficients from a weighted general linear model (GLM), defined separately for each pair of seed areas, modeling the association between their BOLD signal time series. Targeting on the two brain networks, we prescribed the two seed regions, posterior cingulate cortex (PCC) and left intraparietal sulcus (IPS), for DMN and DAN, respectively. Group analysis was carried out using a second-level GLM, in which voxel-level hypotheses were evaluated using multivariate parametric statistics with random effects across subjects and sample covariance estimation. Inferences were performed at the level of individual clusters (groups of contiguous voxels), based on parametric statistics from Random Field theory. Results were presented using an FDR-corrected *p* < 0.05 with cluster-size (k) thresholds (k_ALFF_ ≥ 77 mm^3^; k_DMN_ ≥ 108 mm^3^; k_DAN_ ≥ 100 mm^3^).

### Gene sequencing and SCFA

Within 3 h following the stool sample collection, bacterial DNA was extracted using the Qiagen DNA Mini Prep kit (Qiagen, Hilden, Germany) and stored at −80°C until further processing. Next, in the Joint Human Biobank at Taipei Medical University, the 16S rRNA gene was analyzed following Illumina’s recommended protocol (Illumina, San Diego, CA, USA). The v3–v4 region of the bacterial 16S rRNA gene was amplified to construct a DNA library. Dual-index tags and Nextera XT sequencing adapters (Illumina, San Diego, CA, USA) were added to the amplicons. The DNA quality and quantity were assessed using a QSep 100 analyzer (BiOptic, Taipei, Taiwan), and high-throughput sequencing was performed on an Illumina MiSeq 2000 sequencer. OTU identification and taxonomy assignment followed the methodology described by Callahan et al. (2016). Sequence resolution and accuracy were improved using the DADA2 R package (v1.14.1). Taxonomy was assigned based on the SILVA database (v138) with a minimum bootstrap confidence level of 80 (Quast et al., 2013). Multiple sequence alignments were performed using DECIPHER (v2.14.0), and a phylogenetic tree was constructed using phangorn (v2.5.5) (Schliep, 2010). The resulting count table, taxonomy assignments, and phylogenetic tree were integrated and visualized using phyloseq (v1.30.0) (McMurdie and Holmes, 2013).

Since the gene sequencing did not encompass other microbes (archaea, fungi, viruses), we refer our findings as gut bacteriome instead of gut microbiome. Gut bacterial community diversity across the three repeated measures (*pre-shift*, *post-shift*, and *recovery*) was assessed using the phyloseq and vegan (v2.5.6) R packages. Alpha diversity indices, including observed OTUs, Shannon diversity index, Simpson diversity index, and Chao1 richness estimator, were calculated to measure richness, evenness, and overall diversity. Statistical comparisons of alpha diversity between groups were conducted using t-tests and Wilcoxon tests. For beta diversity, principal coordinate analysis (PCoA) based on UniFrac distances was performed using Adonis from the vegan package to assess differences in OTU composition across the time points. Differential abundance analyses for individual OTUs were conducted using Kruskal–Wallis and Wilcoxon tests with UniFrac (v1.1), with significance set at *p* < 0.05 (Chen et al., 2012).

For SCFA, we homogenized 50 mg of human fecal sample in 0.5 mL of phosphate-buffered saline (0.5%) and extracted the supernatant. Subsequently, we added an equal volume of ethyl acetate (EA) and mixed thoroughly, followed by centrifugation at 14,800 rpm for 15 min. We injected 1 μL the supernatant into Gas Chromatography–Mass Spectrometry (GC/MS) for SCFA analysis, leading to determination of the contents, including acetate, propionate, and butyrate.

### Statistical analyses

R software (4.3.3) and R Studio (2023.12.1 + 402) were used for data analysis after the ROI extraction. We performed one-way repeated-measure analysis of variance (ANOVA) for between-timepoint comparison on cognition, brain functions, and gut bacteriome with post-hoc tests for pairwise comparison, where the statistical significance was set as *p* < 0.05 with Bonferroni correction. Furthermore, to investigate the gut-brain relationship, the Pearson correlation analysis across the 3 repeated measures were conducted between the indices of brain function and gut bacteriome, with false discovery rate correction (FDR-corrected *p* < 0.05).

## Results

### Duration of night shifts, sleep metrics, and cognitive functions

**Figure 1:**
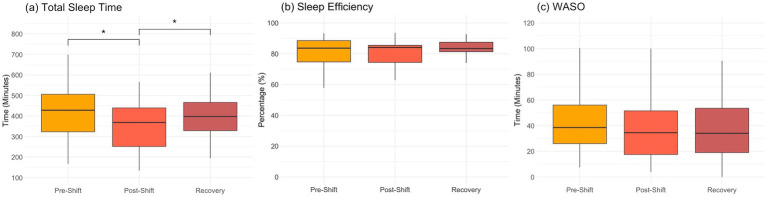
Sleep metrics over the consecutive night shifts (before, after and recovery) in medical staff (*n* = 10). **(A)** total sleep time; **(B)** sleep efficiency; **(C)** wake after sleep onset (WASO). One-way repeated-measure ANOVA and post-hoc tests with Bonferroni correction are used for cross-session comparisons (*p < 0.05 in *post-hoc* tests).

Ten out of 15 recruited participants completed the entire procedure of three repeated measures and achieved all the requirements in night shifts. The averaged time spans of the shift-work and recovery days were listed in Table 1. The sleep metrics during the experimental procedure was shown in Figure 1. After the shift work, it was prominent that the TST reduced from the original 420.6 ± 123.6 min to 350.5 ± 111.1 min, and rebounded back to 440.7 ± 185.4 min in recovery days, indicating a consequence of partial sleep deprivation during the shift work (*F*_2,145_ = 5.12, *p* = 0.007). However, the SE did not show significant changes along the three measures (*F*_2,145_ = 0.53, *p* = 0.59), so as the WASO (*F*_2,145_ = 0.21, *p* = 0.81). Most of the assessed cognitive functions (i.e., memory function, psychomotor ability, and emotion recognition) did not show significant time effects (*F*_2,27_ < 2.61, *p* > 0.10) except the MTT task in the executive function. The MTT exhibited the reduced reaction time in *recovery* session as compared with that in *pre-shift* (Bonferroni-corrected *p* < 0.002, Supplementary Figure S1).

**Table 1:** Demographics and questionnaires from the experimental group.

Demographics and questionnaires	(mean ± s.d.)
Recruited sample size	15
Final sample size (All fMRI sessions completed)	10
Sex	3 male, 7 female
Age	25.67 ± 4.27 years
Time span before night shift	6.22 ± 1.72 days
Time span after night shift	5.56 ± 1.01 days
Time span of recovery	4.11 ± 1.36 days

### Brain functions after night shifts and recovery

**Figure 2:**
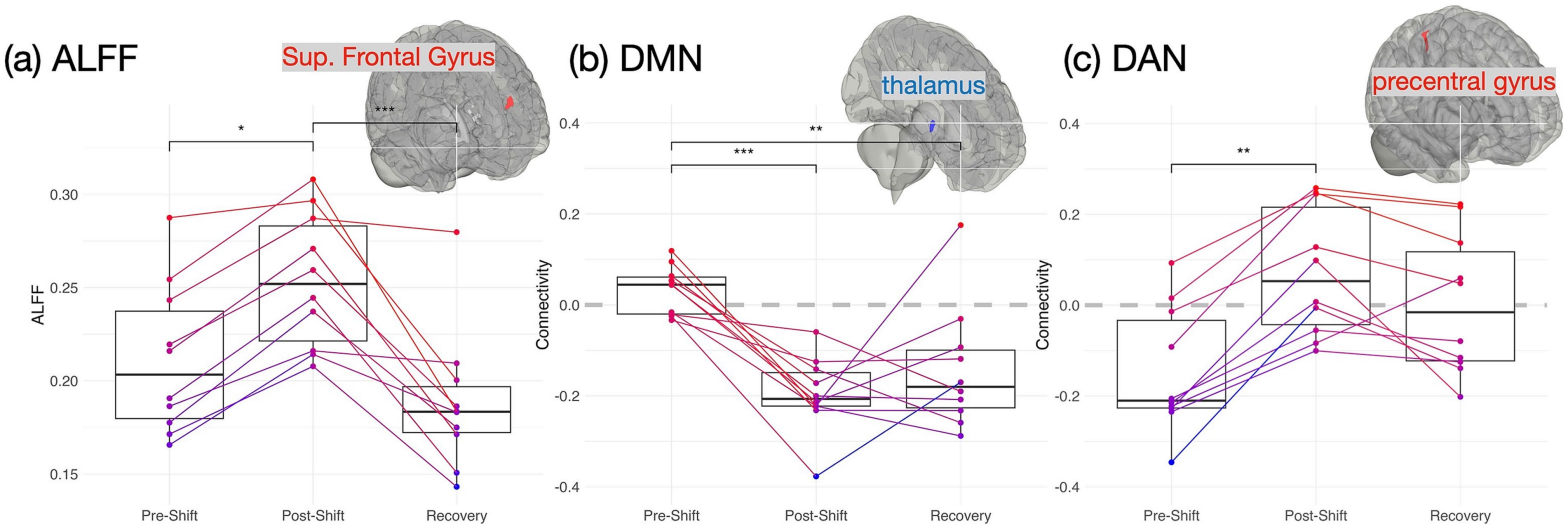
Brain functionality over the consecutive night shifts (before, after and recovery) in medical staff. **(A)** ALFF; **(B)** FC of default-mode network (DMN, seeding at posterior cingulate cortex) connecting to bilateral thalamus; **(C)** FC of dorsal attention network (DAN, seeding at left intra-parietal sulcus) connecting to the precentral gyrus. One-way repeated-measure ANOVA and post-hoc tests with Bonferroni correction are used for cross-session comparisons. (*p < 0.05, **p < 0.01, and ***p < 0.001 in *post-hoc* tests).

To evaluate the night-shift effects on brain functionality, we specifically assessed the ALFF across the 3 repeated measures (Figure 2A). The superior frontal gyrus (SFG) exhibited a significant time effect on ALFF (*F*_1.2,16.5_ = 19.16, *p* < 0.001, *η_p_^2^* = 0.36), where the *post hoc* tests, Bonferroni adjusted, revealed that the medical staff had elevated ALFF after shift work (*p* = 0.022) but returned to the baseline after *recovery* (*p* < 0.001), while no significant differences was found between the *pre-shift* and *recovery* sessions (*p* = 0.21). Figures 2B,C exhibit the FC changes across the 3 measures over the shift work, targeting on the two networks: DMN and DAN, respectively. In DMN (Figure 2B), we observed the time effect on the FC between PCC and thalamus (FC_PCC-thalamus_, *F*_2,27_ = 16.63, *p* < 0.001, *η_p_^2^* = 0.53), indicating that a significant reduction of FC_PCC-thalamus_ in the *post-shift* session (*pre-shift* vs. *post-shift*: *p* < 0.001) remained low in the recovery days (*post-shift* vs. *recovery*: *p* = 0.65). In DAN (Figure 2C), the time effect was found on the FC between the IPS and the precentral gyrus (PreCG) (*F*_2,27_ = 24.79, *p* < 0.001, *η_p_^2^* = 0.31), indicating an elevated FC_IPS-PreCG_ in the *post-shift* session (*pre-shift* vs. *post-shift*: *p* < 0.01).

### Gut bacterial compositions and SCFA after night shifts and recovery

**Figure 3:**
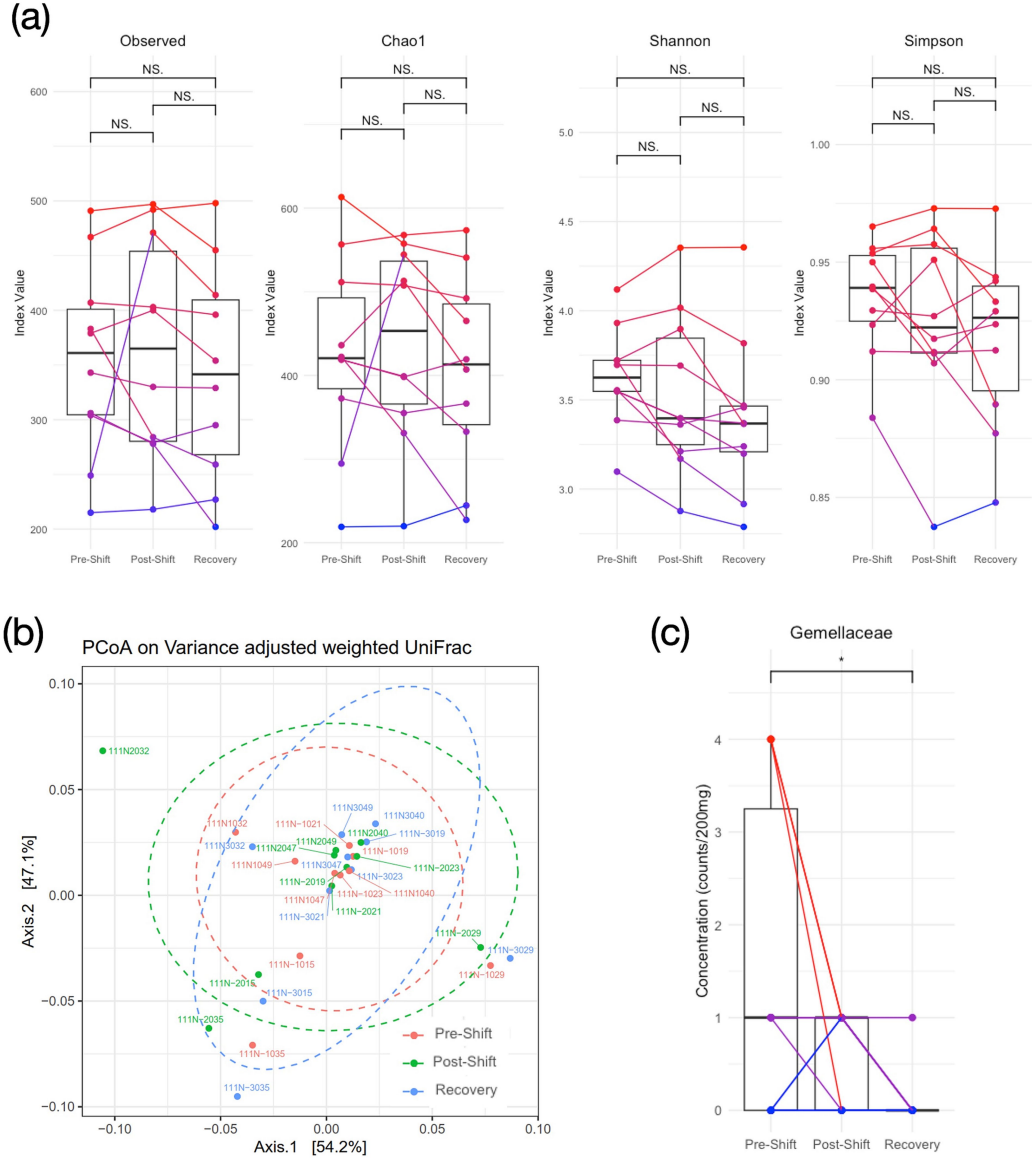
Diversity of gut bacteriome over the consecutive night shifts (before, after and recovery) in medical staff. **(A)** Alpha diversity; **(B)** Beta diversity based on weighted UniFrac PCoA plots; **(C)** Concentration of Gemellaceae in the Family level. One-way repeated-measure ANOVA and post-hoc tests with Bonferroni correction are used for cross-session comparisons. (NS: *none significance*, and *p < 0.05 in *post-hoc* tests).

We estimated the gut bacterial composition across the repeated measures through the analysis of alpha and beta diversity. Figure 3A exhibits that no significant time effect was presented among the number of Observed species (*F*_2,27_ = 0.54, *p* = 0.59), the Chao1 index (*F*_2,27_ = 0.79, *p* = 0.47), and the Simpson diversity (*F*_2,27_ = 2.58, *p* = 0.10). Only the Shannon diversity exhibited significant variation (*F*_2,27_ = 4.95, *p* = 0.019), indicating a lowered diversity after night shifts and recovery; however, no significant difference was shown in the follow-up *post hoc* tests with Bonferroni correction. In beta diversity, the principal coordinate analysis (PCoA) based on variance adjusted with weighted UniFrac index (Figure 3B) exhibited no significant difference in gut bacterial composition across repeated measures, and the relative abundance of the top 200 in both Phylum and Family levels also exhibited insignificant difference across sessions (Supplementary Figure S3). We subsequently estimated bacterial phyla abundances across three time points, but still no significant time effects were observed in all tested bacterial phyla, including *Actinobacteriota*, *Bacteroidota*, *Campilobacterota*, *Desulfobacterota*, *Firmicutes*, *Fusobacteriota*, *Patescibacteria*, *Proteobacteria*, *Synergistota*, and *Verrucomicrobiota* (Supplementary Figure S2). Notably, the *Firmicutes*/*Bacteroidota* ratio (F/B ratio) also demonstrated non-significant time effect (*F*_2,27_ = 1.58, *p* = 0.23, and *η_p_^2^* = 0.03). Across the 56 families, only the *Gemellaceae* showed significant time effect (*F*_2,27_ = 4.40, *p* = 0.028) with significant post-hoc comparison (*pre-shift* vs. *recovery*: *p* = 0.023, Figure 3C). In SCFA, Supplementary Figure S4 shows the boxplots of SCFAs across the 3 repeated measures, and no significant changes was found for all 3 types of SCFA (*F*_2,24_ < 1.92, *p* > 0.18).

### Gut-brain relations after night shifts and recovery

**Figure 4:**
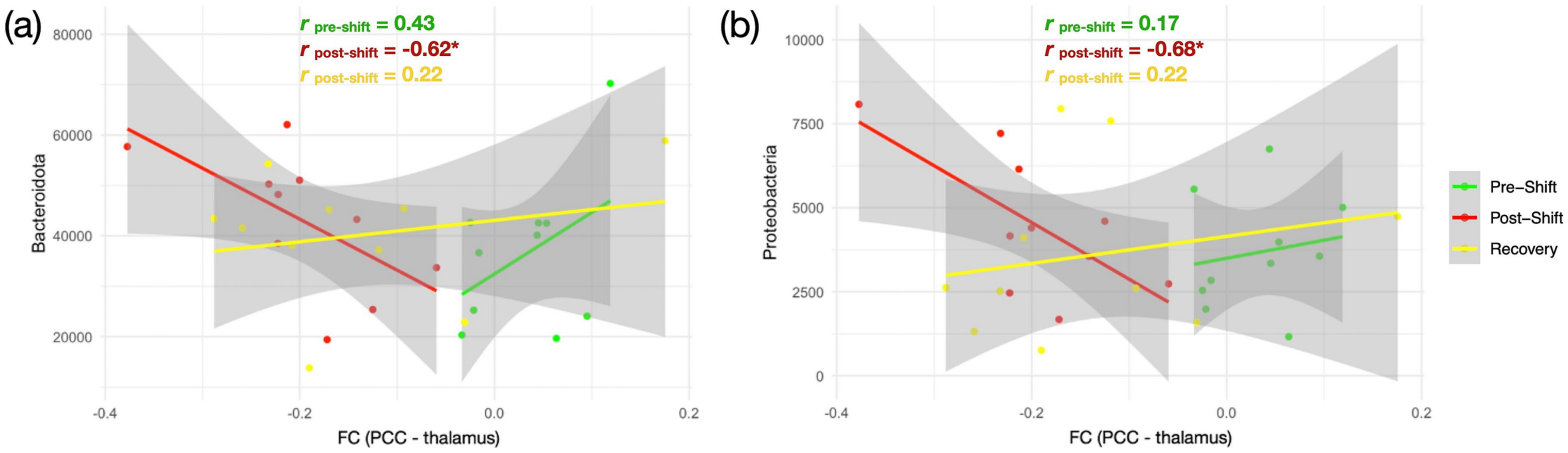
Relationship between brain FC and gut bacteriome over the consecutive night shifts in medical staff. **(A)** Association between *FC_PCC-thalamus_* and *Bacteroidota*; **(B)** Association between *FC_PCC-thalamus_* and *Proteobacteria*. The three repeated measures are marked in different colors—pre-shift (green), post-shift (red), and recovery (yellow). Pearson correlation analysis is conducted across the 3 sessions with false discovery rate correction (*FDR-corrected *p* < 0.05).

The correlation between the FC and gut bacteriome composition were utilized to represent the gut-brain associations across the 3 measures over the shift-work period. No significant difference was found between brain FC and alpha diversity indices in the correlation analysis with FDR correction (Z < 1.96, *p* > 0.05). In assessing the bacterial phyla abundance, we noticed that the original positive relationship between FC_PCC-thalamus_ and *Bacteroidota* (*r* = 0.43) turned into a negative correlation during the *post-shift* (*r* = −0.62), and returned back to a positive correlation (*r* = 0.22) in the *recovery* session (Figure 4A). Similarly, the relationship between the FC_PCC-thalamus_ and *Proteobacteria* also exhibited the positive–negative–positive associations across the 3 time points (*r_pre-shift_*: *r_post-shift:_ r_post-rest_* = 0.17: −0.68: 0.22) (Figure 4B).

## Discussion

We investigated whether the night-shift workstyle impacts the cognition, brain function, and gut bacteriome among Taiwanese medical professionals along the 3 repeated measures (*pre-shift*, *post-shift*, and *recovery*). Partial sleep deprivation was noticed after the 5-day night shifts (Figure 1). The fMRI results disclosed that the brain functionality (*ALFF, DAN and DMN*) was changed after the night-shift work. However, the assessments in the gut bacteriome (alpha/beta diversity, phyla abundance and SCFA) did not show significant changes along the 3 time points, so as most the cognition domains (except *executive function*, Supplementary Figure S1). These observations imply that the circadian misalignments due to night shifts showed profound and immediate impacts on the brain functionality, but the majority of cognition and gut bacteriome were not swiftly affected among the medical professionals. At last, after recovery from the night-shift works, the ALFF indeed recovered back to the *pre-shift* state in the *recovery* session (Figure 2A), and so as the gut-brain relationships (Figure 4). The only index remained impaired in the *recovery* session was the FC_PCC-thalamus_ of DMN (Figure 2B), implying that more than 4 days of circadian realignment was insufficient to provide a full recovery on specific brain functionality.

### Brain functional alterations after night shift

ALFF is one frequency-specific index to represent the amplitude of spontaneous brain activity (Yang et al., 2007), through which a recent study disclosed a frontal ALFF reduction among nurses working long-term shifts (Dong et al., 2024). Previous studies also disclosed that ALFF or fractional ALFF (fALFF) in the frontal lobe could change with the sleep deprivation or insomnia. For example, Wang et al. demonstrated reduced ALFF in the middle frontal gyrus under a 24-h total sleep deprivation in healthy young adults (Wang et al., 2016), but Nechifor et al. exhibited an elevated fALFF in the SFG after a partial sleep deprivation (Nechifor et al., 2020). In the previous work of paradoxical insomnia, we reported an elevated ALFF in the SFG for those who had sleep-state misperception (Hsiao et al., 2018), denoting a hyperarousal in SFG. In the current study, we detected another scenario of enhanced ALFF under the night-shift workstyle, which may link to the frontal hyperarousal under the involuntary sustenance of wakefulness during night shifts. However, different from Wang’s observation of reduced ALFF, we speculated that the ALFF changes in the partial sleep deprivation (e.g., after night shifts in this study) was distinctive to those in the total sleep deprivation. An additional correlation analysis (Supplementary Figure S5) illustrated that the ALFF_SFG_ exhibited positive correlations with PSQI total scores (*r* > 0.31) across the 3 measures, especially significant in the *recovery* session (*r* = 0.66, *p* = 0.038). Further investigations are warranted to disclose the ALFF alterations in sleep deprivation and shift work.

FC_DMN_ and FC_DAN_ are associated with the network functions of self-referential and sustained attention, respectively. Literature reported that the shift workers had dysfunctions of both networks, leading to cognitive impairments, especially in the sustained attention (Chellappa et al., 2018; Zhao et al., 2023; Dong et al., 2024). The rationale of network selection leads us to the current findings that the inter-network connections of DMN and DAN were indeed impacted after the consecutive night shifts and they did not return to the baseline even after 4 recovery days to their normal circadian rhythms. Compared with the quick response of ALFF, the FC_IPS-PreCG_ of DAN in the *recovery* session might be half way returning to the baseline, leading to insignificant results compared to the other two sessions (Figure 2C). Relatively, the recovery speed of FC_PCC-thalamus_ (DMN) was even more sluggish where the aversive impact by the night-shift work sustained after multiple recovery days (Figure 2B). Specifically, the functionality of FC_PCC-thalamus_ was referred to the self-awareness. Boveroux et al. conducted the propofol-induced anesthesia associated with the reduction of FC_PCC-thalamus_ (Boveroux et al., 2010), and our previous work also illustrated the relation between the reduced FC_PCC-thalamus_ and the pre-sleep fatigue (Tsai et al., 2014). Meanwhile, the elevated FC_IPS-PreCG_ could be associated with a higher anxiety level (Huang et al., 2021) or with a sleep deprivation condition (Kong et al., 2018), where it could be intuitively linked to a higher vigilance on their motor function during night shifts. Altogether, the dynamic pattern of DAN/DMN connectivity in medical professionals could imply an enhanced anxiety condition, ready to handle plausible medical emergencies, while the consciousness is dissipating during the night-shift work, and such intense mental process did not return even after 4 days of rest.

One concern is raised that the changes of brain functional indices may originate from the instability over the three consecutive MRI measures. To evaluate the stability of the three functional indices without the circadian misalignment, we applied the same imaging protocol to additional seven young participants with regular sleep–wake schedule over 10-day time span. Based on repeated-measure ANOVA, the three functional indices of the control group exhibited stability over the observable time window. Please see Supplementary Figure S6 for statistical details.

### Unaffected cognition and gut bacteriome after night shift

Most of the cognitive tasks tested remained unaffected across the three time points, in contrast to reports in the literature (Rajaratnam et al., 2013; Kecklund and Axelsson, 2016). The unusual observations imply that the Taiwanese medical professionals adapted to bear burden to prevent from medical error, even under consecutive night shifts. One study with a similar design of consecutive 4-night shifts disclosed that the cognitive impairments occurred on the first night of the night shifts, but the nurses gradually regained their cognitive performances in the nocturnal worktime (Chang et al., 2017). This performance adaptation could be one reason for the resilience to the circadian misalignment among the medical personnel. On the other hand, the other possible reason would be the chronic effect of shift work on cognition, especially for the medical personnel working in a repetitive shift schedule. Marquié et al. (2015) reported that the recovery of cognitive function occurs around 5 years after the cessation of any form of shift work, which implies that the medical shift workers may experience consistent and sustained cognitive decline due to their workstyle. Of course, the last influencing factor in the current study would be the limited sample size; therefore, further studies are warranted to prove whether the cognitive functions are affected by shift works.

Additional issue beyond our expectation would be the unchanged gut bacteriome composition across repeated measures. Literature has shown that circadian dysrhythmia, such as the night shift, negatively influences microbiota communities in the gastrointestinal tract, potentially disrupting energy homeostasis, activating pro-inflammatory pathways, and inducing systemic metabolic syndrome in high-risk populations (Bishehsari et al., 2020). Nevertheless, inconsistent with the speculation, the unaffected gut bacteriome in the current work could be due to insufficient time duration to induce the biological dysbiosis of gut bacteriome. Previously, Liu et al. (2020) conducted similar repeated-measure design on healthy adults and found that acute sleep–wake cycle shift did not cause influences on the alpha and beta diversity of the gut microbiota. The finding of no overt changes in gut microbial abundance and diversity following circadian or sleep shifts was consistent with previous studies (Benedict et al., 2016; Zhang et al., 2017), indicating a resilience of microbial eubiosis in human studies. Interestingly, in rodent models, the sleep deprivation could cause detrimental effects on the gut microbiota compositions. Altaha et al. (2022) conducted a 24-h central clock disruption in mice to mimic shift work and found the dysfunctional arrhythmicity of microbial functions. We conducted a 72-h total sleep deprivation in C57BL/6 J mice and found the dramatic reduction of alpha and beta diversity of microbiota, along with the body-weight decline and increased anxiety behavior (Yang et al., 2023). However, while we conducted a partial sleep deprivation (10 h per day, 8 am to 6 pm) extended to 28 consecutive days, the alpha diversity index of Shannon and Simpson did not show much disparities as compared to the control group (Tung et al., 2024), indicating different mechanisms affecting microbiota between the chronic and acute sleep deprivation.

The *Gemellaceae* is the only family that showed time effect among the 56-family analysis (Figure 3C). Level of *Gemellaceae* significantly decreased after the *recovery* session compared with the *pre-shift* session. Komanduri et al. (2021) reported that the concentration of *Gemellaceae* positively correlated with speed of memory and power of attention in a human aging study, and significant reduction of *Gemellaceae* were found with frailty in neurodegenerative aging (Borrego-Ruiz and Borrego, 2024). Considering the *Gemellaceae* is a family member of *Firmicutes*, a dominant phylum in most human gut environment, it may play an important role in mediating metabolism through regulating SCFA production (Houtman et al., 2022). However, singular family changes seemed unable to change the equilibrium status in the phylum level, based on the insignificant distinctions in *Firmicutes* after the night shifts.

### Night shift changed the gut-brain axis

This is a pioneer study presenting the dynamic neuroimaging changes of gut-brain association. Although we did not find the significant associations between microbial diversity and brain connectivity across the 3 measures of shift work, the correlation alterations between the FC_DMN_ and *Bacteroidota* and *Proteobacteria* (Figure 4) demonstrated the gut-brain association could change after the circadian realignments within the time span of a week. Since FC_PCC-thalamus_ possibly associates with the declined consciousness, the negative correlation in post-shift (red line in Figure 4A) indicates a higher abundance of *Bacteroidota* and *Proteobacteria* in response to the progressively decreased FC_PCC-thalamus_. This could be a mechanism originated from the brain malfunction after consecutive night shifts, propagating to alter the gut bacteriome as a compensatory role in the gut-brain axis. However, this effect is supposed to be a one-way direction from brain to gut, because the *Bacteroidota* and *Proteobacteria* might not accumulate sufficiently over the shift-work duration to feedback to the brain in the current design, evidenced by the unchanged SCFA (Supplementary Figure S4). In the *recovery* session, the brain-bacteriome association returned back to a positive correlation, even though the FC_PCC-thalamus_ remained in a low level.

### Limitations

In the current study, the inability to control the food habit was the major confounds affecting our results. Literature has shown that various dietary choices have significant impacts on the stability and functionality of the microbial community (Ross et al., 2024), so the control of dietary habit may alleviate the confounding factor. However, under the high-stress workload in night shifts, the recruited participants declined to comply with the request of maintaining consistent dietary habit. Considering an enormous between-subject variability in food habits, it is suggested to further record the food diary as the covariates for further investigations.

This study contained several limitations. First, the insignificant findings of cognition and gut bacteria may come from the small sample size in statistics, because the arduous procedures in fMRI, fecal-sample collection and neuropsychological assessments refrained the participating willingness of medical shift workers. We plan to enlarge the sample size in the near future to further illustrate the relationship between gut and brain. Second, from a perspective of clinical practices, it is essential to consider the length of shift work experience, given the diversity of shift work schedules and individual experiences. During our data collection, we observed large variability in shift patterns across different hospitals and departments around Taipei area. Distinctive combination of day shifts, night shifts, and days off can present varying levels of fatigue and recovery. This variability may contribute to discrepancies between our results and those of previous rodent trials conducted under strict control. Third, the senior medical staff members are not required to undertake numerous overnight shifts in Taiwan, stemming from a collegial understanding of their family responsibilities. Therefore, most late-night shifts are typically assigned to less experienced, junior, unmarried medical personnel, preserving more daytime working hours for senior team members. Such medical culture led to a potential difficulty for recruiting senior medical staff as the participant in the repeated-measure design over a night-shift workstyle.

## Conclusion

Medical professionals in Taiwan medical fields commonly experience a regular night-shift rotations, where such regular circadian misalignment may deteriorate cognitive performances but their biological mechanism remained elusive. Hence, we hypothesized that the night shift work may perturb the medical shift workers’ brain function, gut bacteriome composition and the gut-brain association. The three repeated measures disclosed that the brain DAN/DMN connectivity changed dynamically, implying the enhanced anxiety at the consciousness dissipation during the night-shift work periods in medical staff. However, the evaluations of the gut bacteriome composition and majority of cognitive functions did not change significantly before and after the night shifts, even after couple days with normal sleep–wake pattern. The unaffected cognition may indicate a good resilience in medical personnel to the night-shift workstyle, and the unchanged gut bacteriome composition may indicate that the five-day night shifts with partial sleep deprivation was insufficient to induce microbial dysbiosis.

## Data Availability

The raw data supporting the conclusions of this article will be made available by the authors, without undue reservation.
