# Supplementary material for: Impacts of night shift on medical professionals: a pilot study of brain connectivity and gut microbiota
Source: Front Neurosci. 2025 Feb 17;19:1503176. doi: 10.3389/fnins.2025.1503176 (PMC11872915; doi:10.3389/fnins.2025.1503176)
Supplement: Supplementary file 1 [file Data_Sheet_1.PDF]

Impacts of Night Shift on Medical Professionals: A Pilot Study of Brain Connectivity and Gut Microbiota

Supplementary Materials

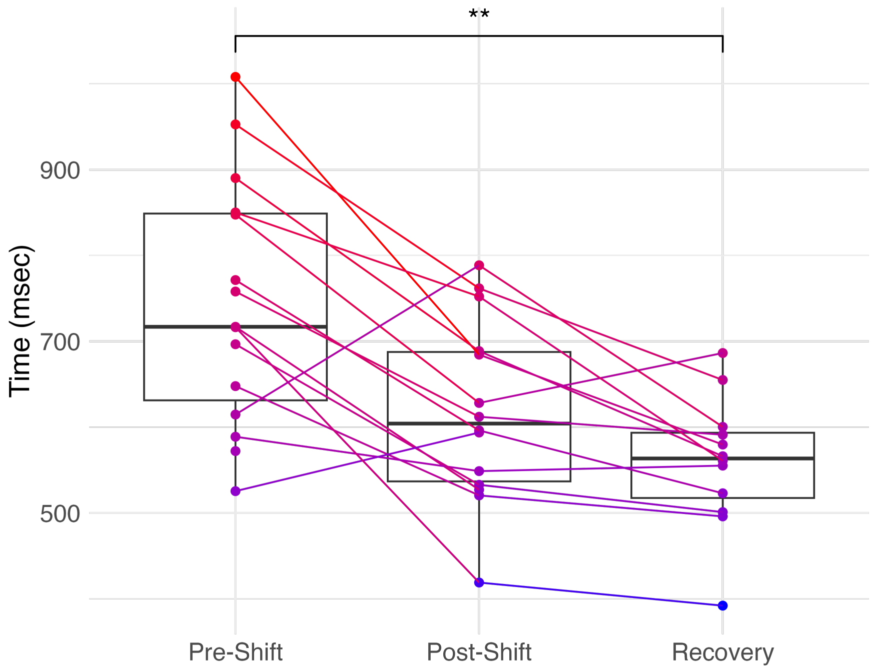

Figure S1. Reaction time in the multitasking test (MTT) over the consecutive night shifts (before, after and recovery) in medical staff. One-way repeated-measure ANOVA and post-hoc tests with Bonferroni correction are used for cross-session comparisons. (**: $p < 0.01$ in *post-hoc* tests).

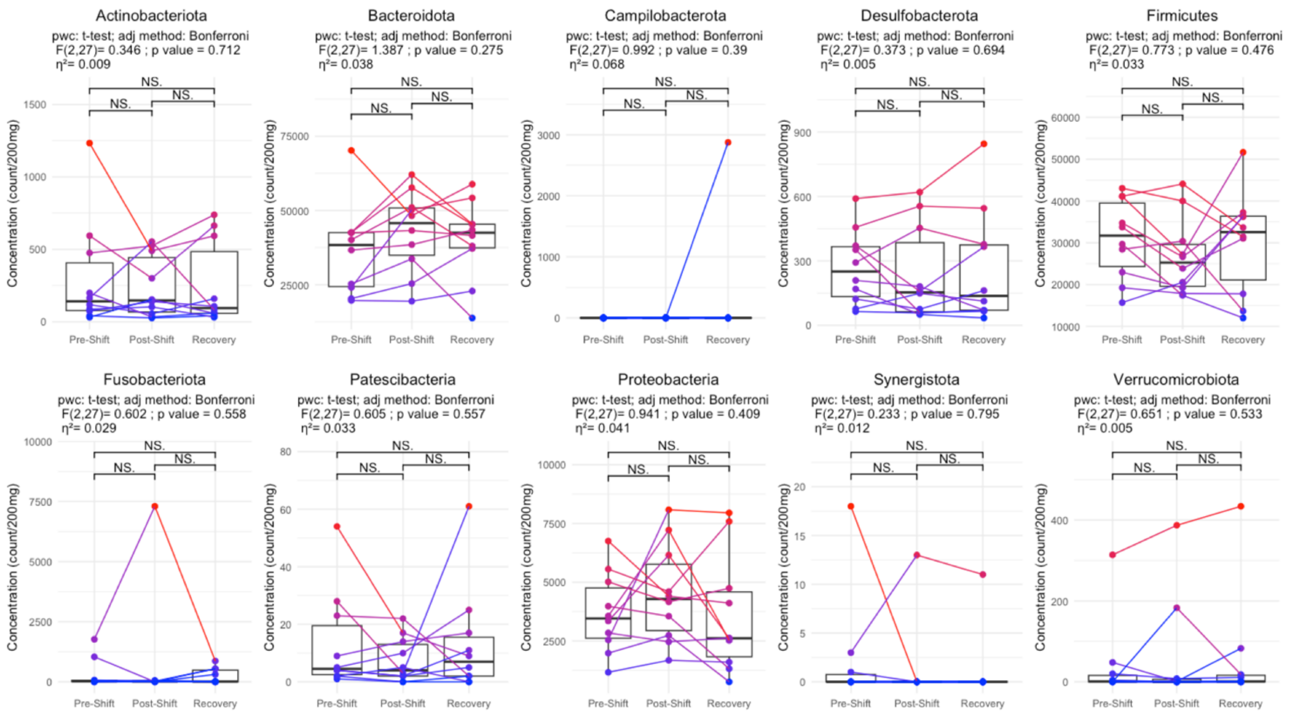

Figure S2. Abundance of gut bacteriome in the phyla level over the consecutive night shifts (before, after and recovery) in medical staff. Ten bacterial phyla were selected for their dominant roles of abundance. One-way repeated-measure ANOVA and post-hoc tests with Bonferroni correction are used for cross-session comparisons. (NS: *none significance* in *post-hoc* tests).

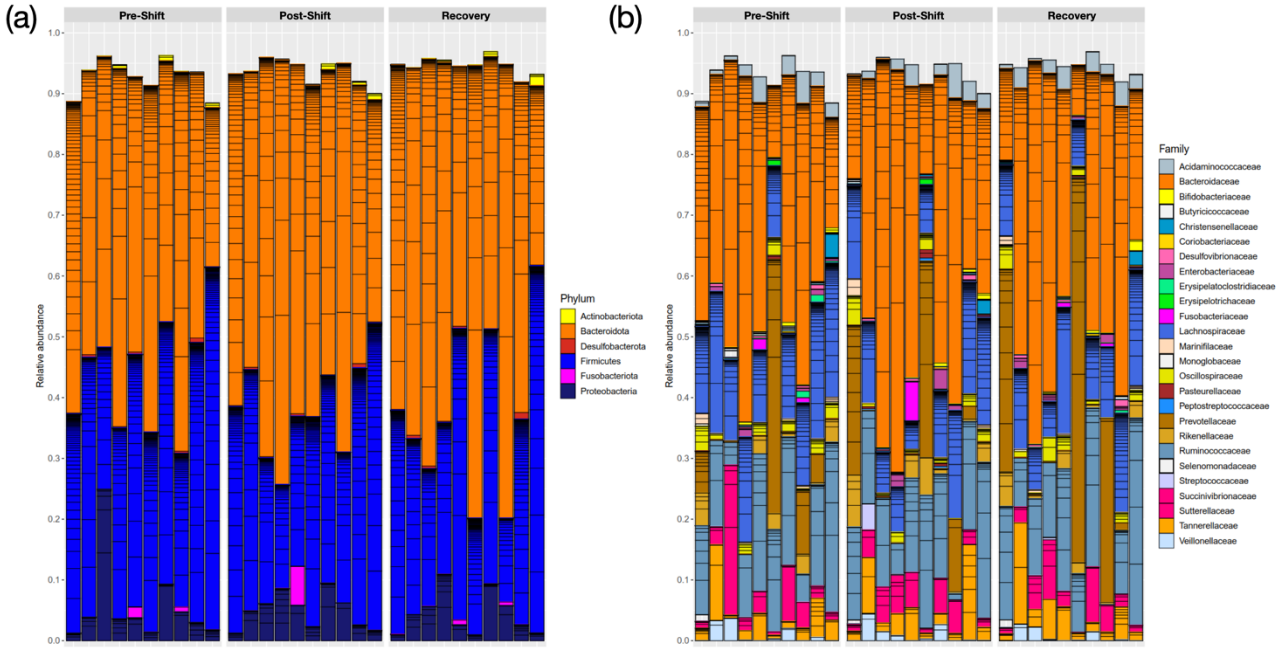

Figure S3. Relative abundance of the top 200 taxa in (a) Phylum and (b) Family level over the consecutive night shifts (before, after and recovery) in medical staff.

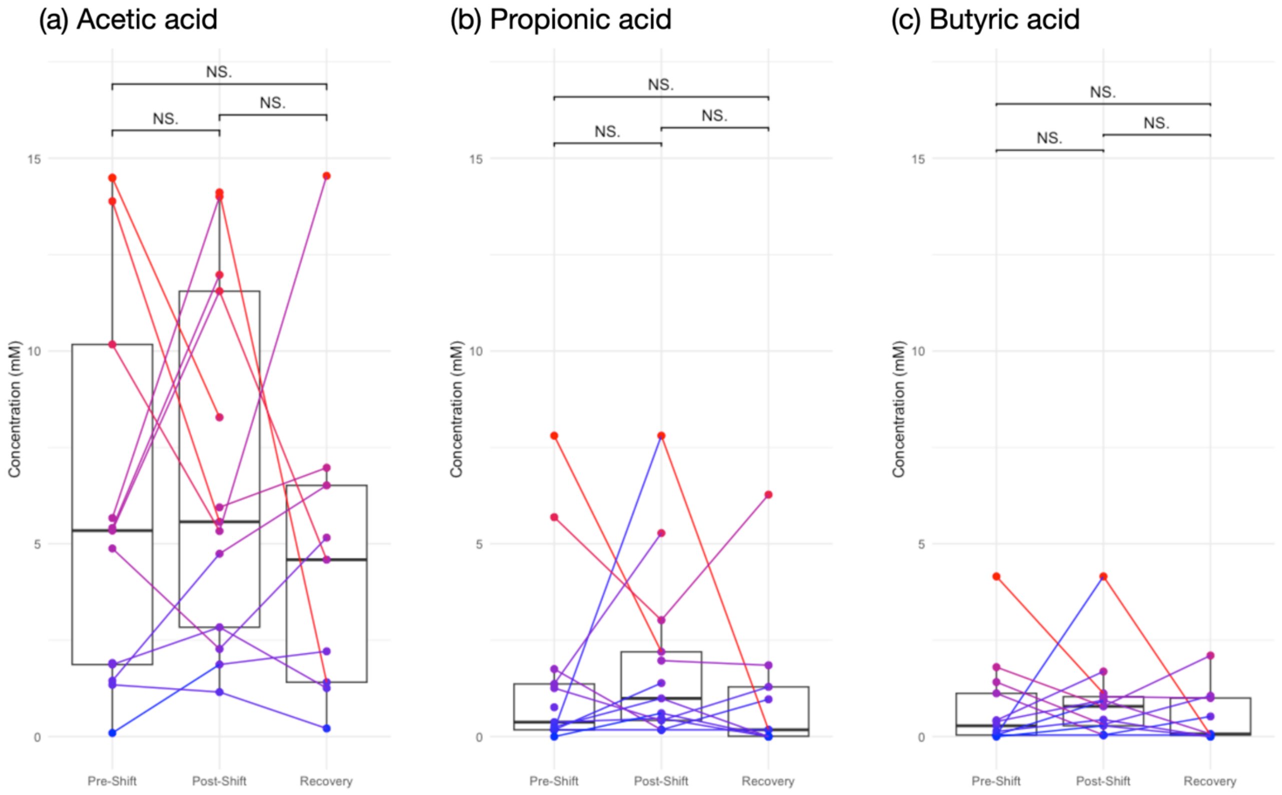

Figure S4. Short-chain fatty acid (SCFA) concentrations over the consecutive night shifts (before, after and recovery) in medical staff. (a) Acetic acid; (b) Propionic acid; (c) Butyric acid. One-way repeated-measure ANOVA and post-hoc tests with Bonferroni correction are used for cross-session comparisons. (NS: *none significance* in *post-hoc* tests).

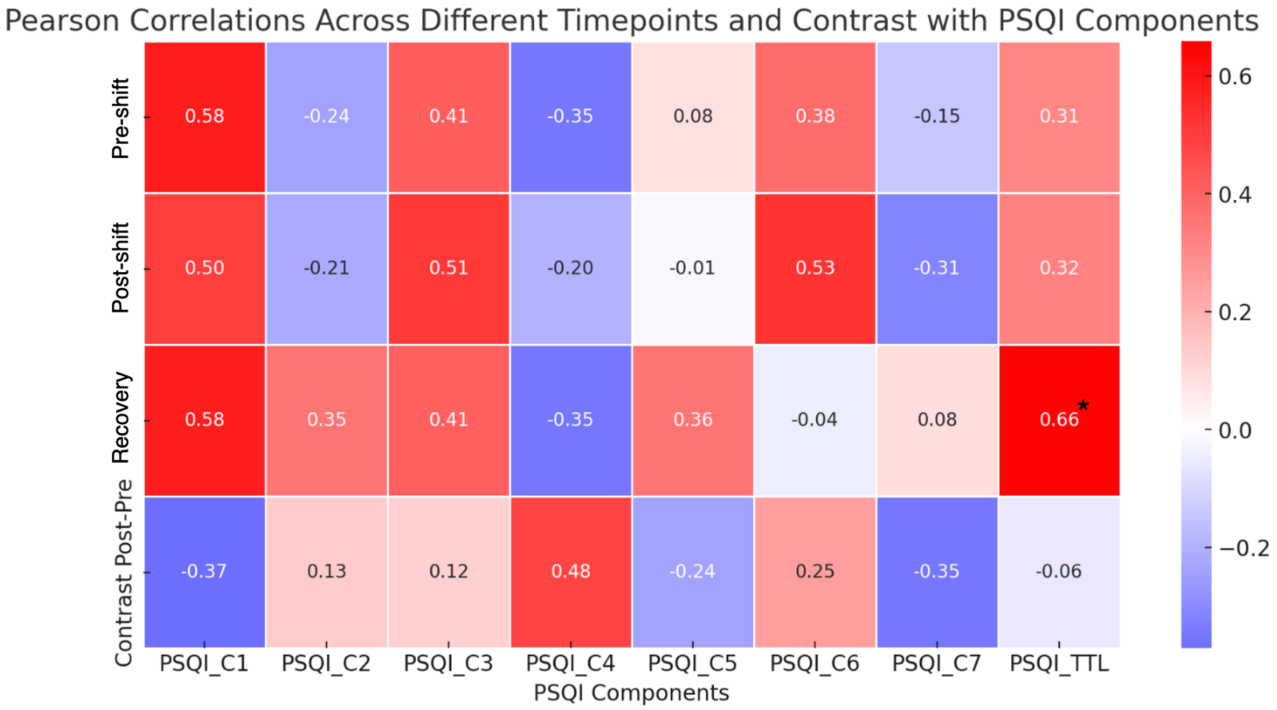

Figure S5. Correlation coefficients between ALFF (SFG) and PSQI (and its sub-scales) over the consecutive night shifts (before, after and recovery) in medical staff. Pearson correlation analyses are conducted with FDR-corrected $p < 0.05$.

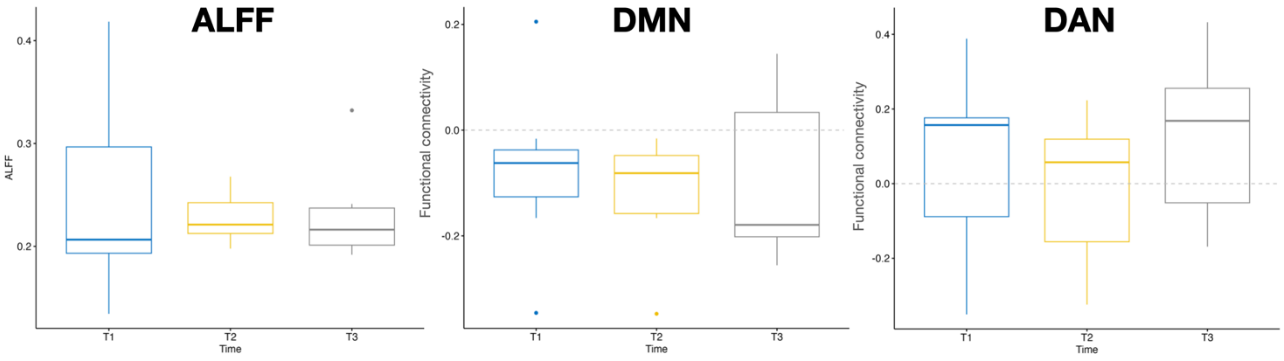

Figure S6. The variations of the three functional indices in the control group (7 normal participants with circadian regularity, mean age ± std = 26.6 ± 1.6) across 3 time points (T1, T2 and T3) over a 10-day time span (T1-T2: 5 days; T2-T3: 4 days). The fMRI data were collected under the same imaging parameters and resembling time gaps among the night-shift schedule. The three functional indices (ALFF, DMN: $FC_{PCC\text{-}thalamus}$, DAN: $FC_{IPS\text{-}PreCG}$) were generated as described in the main text. One-way repeated-measure ANOVA revealed insignificant results among the 7 normal participants (ALFF: $F_{2,12} = 0.19$, $p = 0.83$, DMN: $F_{2,12} = 0.21$, $p = 0.82$, DAN: $F_{2,12} = 1.02$, $p = 0.39$), providing the evidence of reliability for the three brain indices from the control group.
